# A Novel Missense Mutation, I890T, in the Pore Region of Cardiac Sodium Channel Causes Brugada Syndrome

**DOI:** 10.1371/journal.pone.0053220

**Published:** 2013-01-07

**Authors:** Anna Tarradas, Elisabet Selga, Pedro Beltran-Alvarez, Alexandra Pérez-Serra, Helena Riuró, Ferran Picó, Anna Iglesias, Oscar Campuzano, Víctor Castro-Urda, Ignacio Fernández-Lozano, Guillermo J. Pérez, Fabiana S. Scornik, Ramon Brugada

**Affiliations:** 1 Cardiovascular Genetics Centre, Institut d’Investigació Biomèdica de Girona (IDIBGi), Girona, Spain; 2 Medical School, Universitat de Girona (UdG), Girona, Spain; 3 Arrhythmia Unit, Hospital Puerta de Hierro, Madrid, Spain; University of Milan, Italy

## Abstract

Brugada syndrome (BrS) is a life-threatening, inherited arrhythmogenic syndrome associated with autosomal dominant mutations in *SCN5A*, the gene encoding the cardiac Na^+^ channel alpha subunit (Na_v_1.5). The aim of this work was to characterize the functional alterations caused by a novel *SCN5A* mutation, I890T, and thus establish whether this mutation is associated with BrS. The mutation was identified by direct sequencing of *SCN5A* from the proband’s DNA. Wild-type (WT) or I890T Na_v_1.5 channels were heterologously expressed in human embryonic kidney cells. Sodium currents were studied using standard whole cell patch-clamp protocols and immunodetection experiments were performed using an antibody against human Na_v_1.5 channel. A marked decrease in current density was observed in cells expressing the I890T channel (from −52.0±6.5 pA/pF, *n* = 15 to −35.9±3.4 pA/pF, *n* = 22, at −20 mV, WT and I890T, respectively). Moreover, a positive shift of the activation curve was identified (*V*_1/2_ = −32.0±0.3 mV, *n* = 18, and −27.3±0.3 mV, *n* = 22, WT and I890T, respectively). No changes between WT and I890T currents were observed in steady-state inactivation, time course of inactivation, slow inactivation or recovery from inactivation parameters. Cell surface protein biotinylation analyses confirmed that Na_v_1.5 channel membrane expression levels were similar in WT and I890T cells. In summary, our data reveal that the I890T mutation, located within the pore of Na_v_1.5, causes an evident loss-of-function of the channel. Thus, the BrS phenotype observed in the proband is most likely due to this mutation.

## Introduction

Alterations of the sodium current (*I*_Na_) in the human heart can lead to diseases responsible for cardiac arrhythmias, such as Brugada Syndrome (BrS) [1]. This syndrome, first described in 1992, is characterized by the presence of ST segment elevation in the right precordial leads (V1–V3) of the electrocardiogram (ECG), without major structural alterations in the heart [2]. The prevalence of BrS is in the range of 1–5 in every 10,000 individuals and is an important cause of Sudden Cardiac Death (SCD) [3].

Since the discovery of the first genetic variation in the cardiac sodium channel gene, *SCN5A*, associated with BrS [4], many studies have classified this syndrome as a genetic disease with autosomal dominant inheritance and incomplete penetrance [5]. It has been demonstrated that mutations in *SCN5A* associated with BrS result in loss-of-function of the current carried by the cardiac type sodium channel (Na_v_1.5) [6]. Different mechanisms are known to produce channel loss-of-function, including reduced expression of the channel in the plasma membrane, changes in the voltage dependence of the channel activation or inactivation, or altered channel kinetics [7]. In addition, mutations in genes other than *SCN5A* have been identified in a low proportion of BrS patients [8].

The Na_v_1.5 protein, with 2016 amino acids and a molecular weight of 227 kDa, consists of four homologous domains (DI-DIV) [9]. Each domain contains six transmembrane segments (S1–S6) linked by intracellular and extracellular loops. S4 segments contain 5 positively charged residues (arginine or lysine) separated by 2 hydrophobic residues, and form the voltage sensor domain of the channel. The pore region of the channel is formed by the interaction among segments S5, S6 and loop S5–S6 of domains DI to DIV [10]. The pore (P)-helices that stabilize the Na^+^ ion in the central cavity are formed by the loops S5–S6 [11].

In the present study, we aimed to characterize the biophysical properties of Na_v_1.5 channels carrying a novel mutation, I890T, in the first P-helix of DII to establish whether this mutation is associated with BrS. We show evidence of loss-of-function of the mutant Na_v_1.5 channel, which is consistent with the patient’s clinical manifestation of BrS.

## Methods

### Ethics Statement

This study was approved by the Ethics Committee of Hospital Josep Trueta (Girona, Spain) and conforms with the principles outlined in the Declaration of Helsinki. All individuals signed a written informed consent to participate in the study.

### Reagents

All reagents were obtained from Sigma-Aldrich (St. Louis, MO, USA), unless stated otherwise.

### Genetic Analysis of *SCN5A*

Total genomic DNA was isolated from blood samples using the Puregene DNA purification Kit (Gentra Systems, Minneapolis, MI, USA). The exons and exon-intron boundaries of *SCN5A* were amplified (Verities PCR, Applied Biosystems, Austin, TX, USA), the PCR products were purified (Exosap-IT, USB, Isogen Life Science, The Netherlands) and they were directly sequenced in both directions (Big Dye Terminator v3.1 cycle sequencing Kit and 3130XL Genetic Analyzer, both from Applied Biosystems). The DNA sequence was compared with the reference sequence NM000335 for *SCN5A* (OMIM601144) (UCSC Genome binformatics [12]/NCBI-Mendelian Inheritance [13]). DNA samples from 300 healthy Spanish individuals (600 alleles) were used as control samples.

### Site-directed Mutagenesis

The wild-type (WT) human *SCN5A* cDNA (Uniprot reference: Q14524) cloned in pcDNA3.1 (a kind gift from Dr. Matteo Vatta, Baylor College of Medicine, Houston, TX, USA.) was used as template to engineer the mutation I890T using the QuikChange Site-Directed Mutagenesis system (Stratagene, La Jolla, CA, USA) and the following primers (mutation underlined):

5′-GCCTTCCTCACCATCTTCCGCATCCTCTGTGGAGAGTGGATCG-3′ and.

5′–GCGGAAGATGGTGAGGAAGGCATGAAAGAAGTCCATCATGTGC-3′.

The resultant construct was directly sequenced to verify the presence of the desired mutation and the absence of additional variations.

pcDNA3.1 included a FLAG tag (sequence: DYKDDDDK between prolines P154 and P155 of *SCN5A*) which has been previously shown not to alter the Na_v_1.5 current properties [14], [15]).

### Cell Culture and Transfection

Human embryonic kidney (HEK) 293 cells, a kind gift from Dr. Miguel Valverde [16], were used as experimental model. Cells were maintained in Dulbecco’s Modified Eagle’s Medium supplemented with 10% Fetal Bovine Serum, 1% antibiotic-antimycotic and 1% Glutamax (all from Invitrogen, Carlsbad, CA, USA) at 37°C and 5% CO_2_.

HEK cells were transiently transfected with 2.9 µg of the *SCN5A* construct, either WT or I890T, using GeneCellin™ Transfection Reagent (BioCellChallenge, Toulon Cedex, France) following the manufacturer’s specifications. Co-transfection with 0.1 µg of a plasmid containing the green fluorescent protein (GFP) gene (a kind gift from Dr. Kirstine Callø, University of Copenhagen, Copenhagen, Denmark) allowed the identification of transfected cells. All experiments were performed 48 hours after transfection.

### Electrophysiological Studies

Sodium currents were measured at room temperature using the standard whole cell patch-clamp technique [17]. Voltage clamp experiments were controlled and analyzed with an Axopatch 200B amplifier and pClamp 10.2/Digidata 1440A acquisition system (Molecular Devices, Sunnyvale, CA, USA) and OriginPro8 software (OriginLab Corporation, Northampton, MA, USA). The bath solution contained (mM): 140 NaCl, 3 KCl, 10 N-2-hydroxyethylpiperazine- N’ -2-ethanesulfonic acid (HEPES), 1.8 CaCl_2_ and 1.2 MgCl_2_ (pH 7.4, NaOH); and the pipette solution (mM): 130 CsCl, 1 Ethylene glycol-bis(2-amino-ethylether)-N,N, N’,N’-tetra-acetic acid (EGTA), 10 HEPES, 10 NaCl and 2 ATP Mg^2+^ (pH 7.2, CsOH). Osmolality was adjusted by the addition of glucose to 326 and 308 mOsm for bath and pipette solution, respectively. Pipettes were pulled from glass capillaries (Brand GMBH+CO KG, Wertheim, Germany) and their resistance ranged from 2.5 to 3.2 MΩ when filled with the internal solution. 80–90% series resistance compensation was used during whole cell measurements. Membrane potentials were not corrected for junction potentials that arose between the pipette and bath solution. Data were filtered at 5 kHz and sampled at 5–20 kHz.

Activation curve data were fitted to a Boltzmann equation, of the form *g* = *g*_max_/(1+ exp(*V*_1/2_−*V*_m_)/*k*), where *g* is the conductance, *g*_max_ the maximum conductance, *V*_m_ is the membrane potential, *V*_1/2_ is the voltage at which half of the channels are activated and *k* is the slope factor. Steady-state inactivation values were fitted to a Boltzmann equation of the form *I* = *I*_max_/(1+ exp(*V*_1/2_−*V*_m_)/*k*), where *I* is the peak current amplitude, *I*_max_ the maximum peak current amplitude, *V*_m_ is the membrane potential, *V*_1/2_ is the voltage at which half of the channels are inactivated, and *k* is the slope factor. The sodium current decay after the peak *I*_Na_ was fitted with a monoexponential function between −40 and −25 mV, and a bi-exponential function between −20 and 20 mV, from where τ fast and τ slow were obtained. Both the slow inactivation and the recovery from inactivation data were fitted to mono-exponential functions, to obtain their respective time constants.

### Cell Surface Protein Biotinylation

Cells were washed with Dulbecco’s Phosphate-Buffered Saline (DPBS) supplemented with 0.9 mM CaCl_2_ and 0.49 mM MgCl_2_ (DPBS^+^) at pH 7.4. Membrane proteins were biotinylated by incubating cells with 1.6–2.5 mg/ml of EZ-link sulfo-NHS-LC-LC-biotin (Pierce, Thermo Scientific, Rockford, IL, USA) in DPBS^+^ for 30 min at 4°C. Cells were then washed 3 times in DPBS^+^ with 100 mM glycine, then with DPBS^+^ containing 20 mM glycine, and scrapped in Triton X-100 lysis buffer (1% Triton X-100, 50 mM Tris/HCl pH 7.4, 150 mM NaCl, 1 mM EDTA and Complete Protease Inhibitor Cocktail (Roche, Madrid, Spain)). Lysates were obtained after 1 h rotating at 4°C. Insoluble materials were removed by centrifugation. Supernatants were incubated with Ultralink Immobilised NeutrAvidin beads (Pierce) overnight at 4°C. The beads were precipitated and washed with Triton X-100 lysis buffer, then in saline solution (5 mM EDTA, 350 mM NaCl and 0.1% TX-100 in DPBS^+^ pH 7.4) and finally in 10 mM Tris/HCl pH 7.4. Precipitated beads were resuspended in SDS-PAGE loading buffer and heated for 5 min at 70°C. Proteins were resolved in 4% SDS-PAGE gels and transferred to PVDF membranes (Millipore, Billerica, MA, USA). Membranes were probed with a rabbit *anti*-human Na_v_1.5 antibody (*anti*-hNa_v_1.5; Alomone Labs, Jerusalem, Israel) at a dilution of 1∶1,000, overnight at 4°C. A secondary horseradish peroxidase-conjugated antibody (Thermo Scientific, Rockford, IL, USA) was used at a dilution of 1∶2,000 for 1 h at room temperature, and signals were detected with the SuperSignal West Femto Chemiluminiscent substrate (Pierce). A mouse antibody against Na^+^/K^+^ ATPase was used as biotinylation control. Protein markers for molecular weights from 10 to 250 kDa (PageRuler™ Plus Prestained Protein Ladder, Thermo Scientific, Rockford, IL, USA) were used as size standards in protein electrophoresis (SDS-PAGE) and Western blotting.

Expression of Na_v_1.5 was quantified using the ImageJ software (National Institute of Health, NIH) available at http://rsb.info.nih.gov/ij. Intensity values for each band were determined as the integrated density (sum of pixel values) within a fixed area. To account for differences of these values between WT and I890T due to loading, I890T intensity values were normalized with the ratio between WT and I890T Na^+^/K^+^ ATPases.

### *In silico* Studies of I890T

The software tools ESyPred3D 1.0 [18], Modeller 9.9 [19] and CPHmodels [20] were used to build a model of the pore module of DII of Na_v_1.5, based on the structure of the bacterial voltage-gated sodium channel (Na_v_ *Ab*) ([11]). The model was constructed as a chimera of Na_v_ *Ab* and Na_v_1.5 as follows: the sequence of S1 to S4, as well as the loop S4–S5, was that of Na_v_ *Ab*; the sequence of S5, loop S5–S6, and S6 was that of DII of Na_v_1.5. No further constraints were defined.

### Statistical Analyses

Results are presented as means ± standard error (SE). Statistical comparisons were performed using the unpaired Student’s t-test. Results are considered statistically significant when *p*<0.05.

## Results

### Identification of I890T, a Novel Na_v_1.5 Channel Mutation

**Figure 1:**
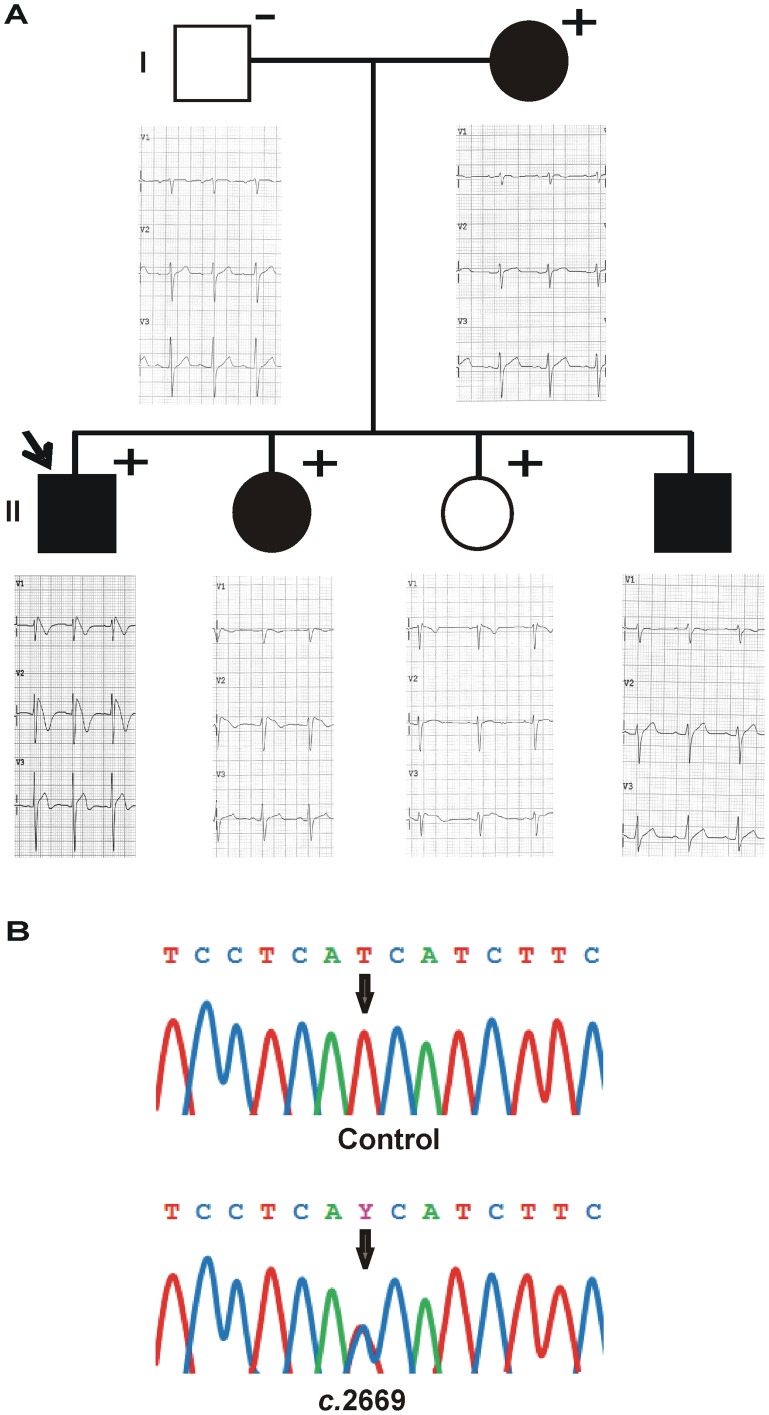
Clinical and genetic characterization of the proband and his family. (A) Family pedigree with corresponding ECGs. Open symbols indicate clinically normal subjects and filled symbols mark clinically affected individuals. Plus signs indicate the carriers of the mutation I890T and minus signs, non-carriers. The arrow identifies the proband. Basal ECG of the proband and ECGs at the time of the ajmaline test of the family members are presented. (B) Detail of the electropherograms obtained after *SCN5A* sequence analysis. The arrow indicates the nucleotide position 2669 of *SCN5A*, where a double peak (T to C heterozygote change, *c*.2669 T>C) was identified in the proband’s DNA.

The proband, a 31-year-old Spanish male, was admitted to the hospital due to the suspicion of BrS during a routine examination. His baseline 12-lead ECG showed a ST segment elevation in leads V1–V3 that strongly suggested BrS type I (Fig. 1A). He had suffered an episode of syncope at the age of 12.

A heterozygous variation (thymine-to-cytosine) at position 2669 of the *SCN5A* gene (*c*.2669 T>C) was identified in the proband’s DNA (Fig. 1B). This base transition leads to an isoleucine-to-threonine substitution at position 890 (*p*.I890T) of the Na_v_1.5 channel. This genetic variation was absent in 600 control alleles of the same ethnic background, and was not found in the Human Gene Mutation Database (HGMD) [21], Ensembl [22], HapMap [23], 1000 genomes project [24] and NHLBI Exome Sequencing Project [25]. The I890T variation in Na_v_1.5 channel thus represents a possible novel mutation causing BrS.

The genetic study of the proband’s family members revealed that two sisters had the I890T mutation (Fig. 1A). A 34-year-old sister presented several episodes of syncope, her baseline ECG was normal, but a ST segment elevation in lead V2 characteristic of BrS was unmasked upon ajmaline challenge, thus confirming BrS. A 18-year-old sister was asymptomatic, had a normal baseline ECG, and presented no alterations after challenge with either flecainide or ajmaline. The proband’s third brother refused to undergo genetic analysis despite having a previous history of syncopes. The proband’s mother carried the I890T mutation. She presented an episode of syncope at age 18, but showed a normal ECG when subjected to drug challenge tests. The proband’s father did not carry the mutation and was asymptomatic, and had a normal ECG after drug provocation test.

To further explore the different clinical phenotypes found among the carriers of the I890T mutation, we looked for other genetic variations in the *SCN5A* gene in the family. We found that the younger sister, who did not present any cardiac abnormalities, carried a non-synonymous polymorphism, *p*.H558R, inherited from the father. This variation was not found in any of the other mutation carriers.

### I890T Markedly Decreases Peak *I*_Na_ and Modifies Na_v_1.5 Channel Activation Kinetics

**Figure 2:**
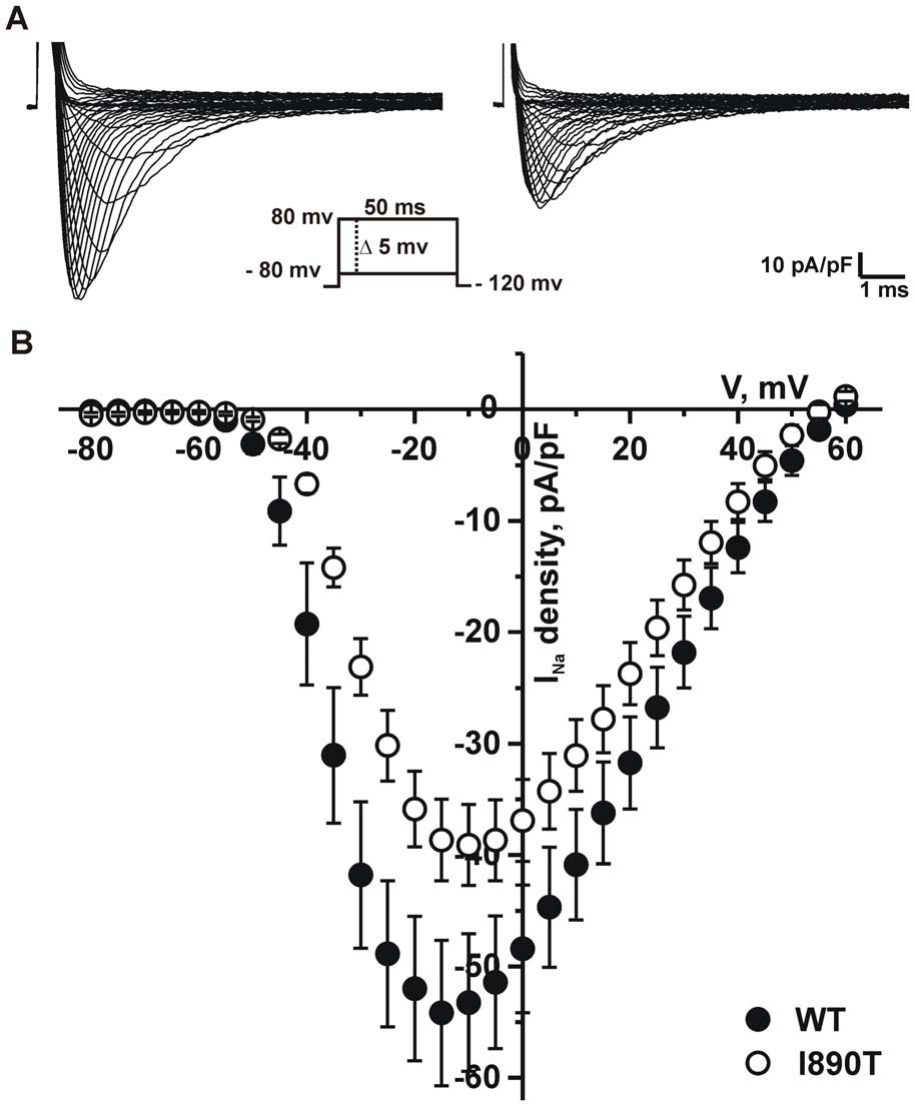
I890T markedly decreases peak *I*_Na_. Voltage dependence of sodium currents measured from WT and I890T cells. Whole cell currents were elicited by depolarizing potentials as shown in the inset. (A) Representative whole cell sodium current density traces recorded from WT and I890T cells. (B) Current-voltage (*I*–*V*) relationship. *I*_Na_ amplitude was normalized to the cell capacitance to obtain current density (*I*_Na_ density) values. Experimental points represent the peak-amplitude of current density at each given voltage, for WT (filled circles) and I890T (open circles). Values are expressed as mean ± SE.

We conducted patch-clamp studies to assess the effect of the mutation I890T on whole cell sodium currents. HEK cells were transfected with either WT or I890T channel constructs (referred to as WT cells and I890T cells, respectively, in the remaining text). Current traces in Figure 2A show that *I*_Na_ is substantially reduced by the mutation I890T. This reduction was confirmed by analysis of peak *I*_Na_ density, which showed a significant decrease in the current-voltage relationship (*I*–*V*) of I890T cells with respect to WT cells (Fig 2B, Table 1).

**Table 1:** Biophysical parameters of WT and I890T channels.

	*I*_Na_ at −20 mV		Activation			Steady-state Inactivation			Slow inactivation		Recovery from inactivation	
	pA/pF	*n*	*V*_1/2_ (mV)	*k*	*n*	*V*_1/2_ (mV)	*k*	*n*	*τ* (ms)	*n*	*τ* (ms)	*n*
**WT**	−52.0±6.5	15	−32.0±0.3	−6.9±0.3	18	−84.9±0.9	−4.9±0.4	10	243.2±39.9	5–14	3.9±0.1	11
**I890T**	−35.9±3.4*	22	−27.3±0.3**	−6.7±0.2	22	−84.2±0.4	−4.9±0.4	15	224.2±35.0	5–19	4.2±0.1	16

Activation and steady-state inactivation parameters were calculated by data fitting to Boltzmann functions (see Methods). *V_1/2_* is the voltage for half-maximal activation or steady-state inactivation and *k* is the slope factor. Slow inactivation and recovery from inactivation data were fitted to mono-exponential functions (see Methods) to obtain the time constant *τ*. Values are expressed mean ± SE. *p<0.05; **p<0.01.

**Figure 3:**
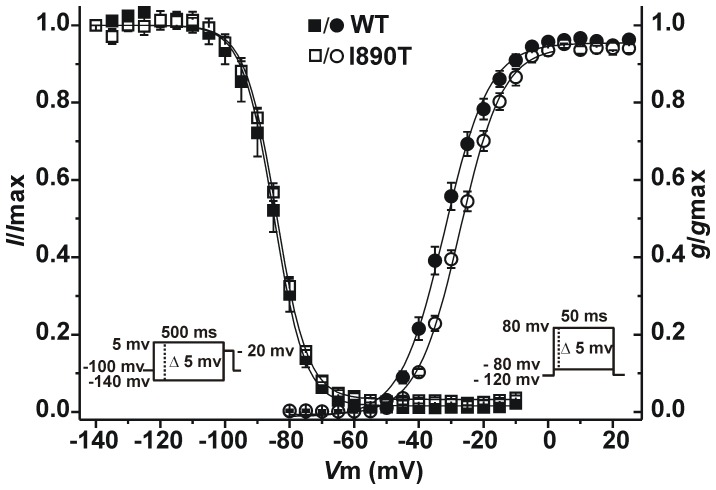
I890T modifies Na_v_1.5 channel activation kinetics. *I*_Na_ voltage-dependence of activation and steady-state inactivation for WT and I890T cells. Conductance values for the activation curve were obtained from the peak current values taken from Figure 2. Symbols represent experimental data plotted against the given depolarizing voltage values for WT (filled circles) and I890T (open circles). Steady-state inactivation protocol is shown in the inset on the left. Relative current values were determined using 500 ms pre-pulses to different potentials followed by a test pulse to −20 mV. Symbols represent experimental data plotted against preconditioning pulse values for WT (filled squares) and I890T (open squares). Values are expressed as mean ± SE. Solid lines represent the Boltzmann fit of the experimental points.

In addition, we observed a positive shift of the activation curve towards more positive potentials (Fig. 3), which further contributes to the loss-of-function of I890T channels. Data fitting to a Boltzmann equation revealed a significant 5 mV shift of *V*_1/2_ in I890T respect to WT cells, whereas no changes were observed in the slope factor (Table 1).

Next, we assessed the voltage-dependence of steady-state inactivation for WT and I890T cells. The mutation I890T did not affect the voltage dependence of channel availability (Fig. 3, Table 1).

**Figure 4:**
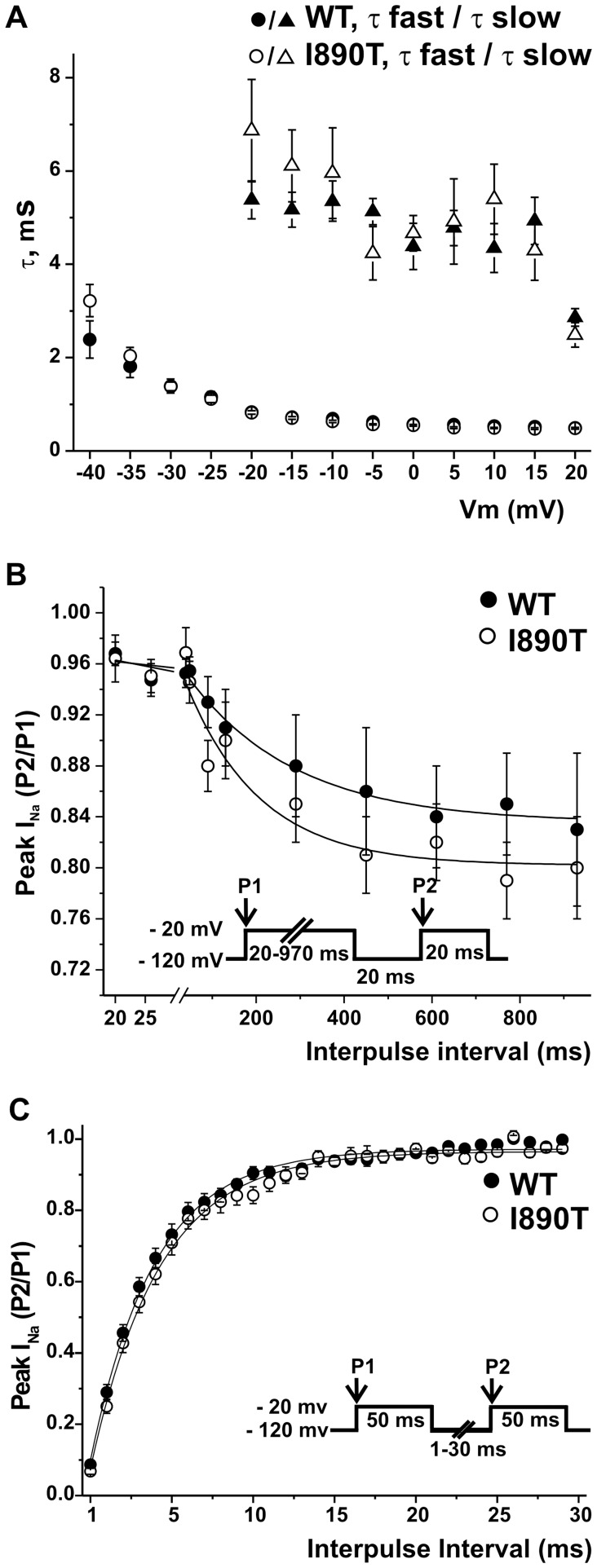
I890T does not affect the time course of inactivation, slow inactivation, or recovery from inactivation. (A) Experimental data obtained for the current-voltage relationship (Fig. 2) was used to determine inactivation time constants in the voltage range between −40 and 20 mV. Current decay after the peak *I*_Na_ was fitted to a mono-exponential function (from −40 to −25 mV) and a bi-exponential function (from −20 to 20 mV), and the resulting time constants (*τ*) were plotted *versus* the applied voltage for WT and I890T. (B) Voltage dependence of slow inactivation for WT and I890T were studied by applying the double protocol pulse shown in the inset. A 20–970 ms conditioning pre-pulse to −20 (P1) was followed by a 20 ms hyperpolarization to −120 mV, to recover fast-inactivated channels, and then a 20 ms test pulse to −20 mV (P2). The peak current ratio P2/P1 was plotted against the P1 prepulse duration, and data was fitted to mono-exponential functions (solid lines). (C) Recovery from inactivation properties for WT and I890T were studied by applying the double pulse protocol shown in the inset. A 50 ms depolarizing pulse to −20 mV (P1) was followed by a hyperpolarizing pulse to −120 mV of increasing duration (1–30 ms), that preceded a test pulse to −20 mV (P2). The P2/P1 ratio values plotted against the recovery interval times were fitted to mono-exponential functions (solid lines). A, B and C: Values are expressed as mean ± SE. Symbols represent values for WT (filled symbols) and I890T (open symbols).

Analysis of the time courses of inactivation, slow inactivation and recovery from inactivation are illustrated in Figure 4. Inactivation time constants were obtained from fitting the time course of the currents elicited with the stimulation protocol used for the *I*–*V* relationship, and plotted as a function of voltage (Fig. 4A). The time constants for I890T and WT currents remained similar at the voltage range analyzed. Double pulse protocols were used to study *I*_Na_ slow inactivation and recovery from inactivation. No differences were found either in the slow inactivation or recovery from inactivation parameters obtained from fitting of data from WT and I890T cells to mono-exponential functions (Fig. 4B and 4C, respectively, and Table 1).

### I890T does not Alter Membrane Expression of Na_v_1.5 Channel

**Figure 5:**
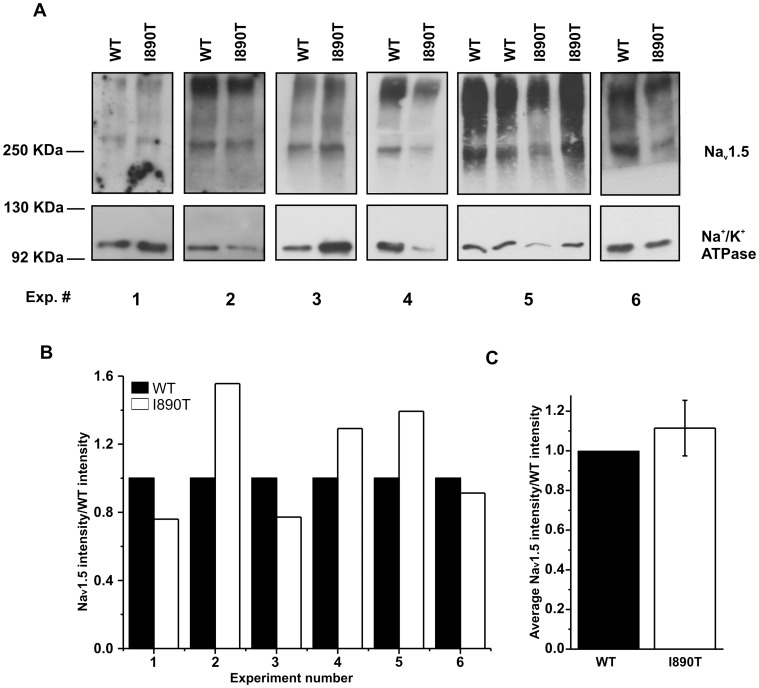
Membrane expression of Na_v_1.5 channel is not affected by I890T. Western blot detection of Na_v_1.5 and Na^+^/K^+^ ATPase proteins performed after cell surface biotinylation from WT and I890T cells. (A) Image shows the bands obtained for Na_v_1.5 and Na^+^/K^+^ ATPase, in 6 independent experiments from cells expressing either WT or I890T. Position of the markers is shown on the left side. Numbers correspond to each experiment. (B) Bar graph depicts the Na_v_1.5 intensity values normalized by the Na_v_1.5 WT intensity values (n = 6). Na_v_1.5 I890T intensity values were previously corrected by multiplying the raw integrated density values by the ratio between the WT and I890T Na^+^/K^+^ ATPase integrated density values. Note that for experiment number 5, the ratio between I890T and WT was calculated from the average of the intensity values obtained for the two samples of each condition. (C) Bar graph shows the average intensity values (expressed as mean ± SE) obtained in (B).

I890T caused a significant decrease in *I*_Na_ (Fig. 2). Thus, we tested whether this change in *I*_Na_ was caused by alterations in Na_v_1.5 expression in the plasma membrane. Figure 5A shows western blot bands obtained after cell surface protein biotinylation from 6 independent experiments. Densitometry analysis of the bands (Fig. 5B and C) did not reveal statistically significant differences between WT and I890T cells (Na_v_1.5 I890T/WT was 1.11±0.14, n = 6).

Taken collectively, our data indicate that the I890T mutation causes a loss-of-function of Na_v_1.5 current by the modification of the biophysical properties intrinsic to channel activity, rather than by impaired expression at the plasma membrane.

### *In silico* Models of the Pore Region of WT and I890T DII of Na_v_1.5

**Figure 6:**
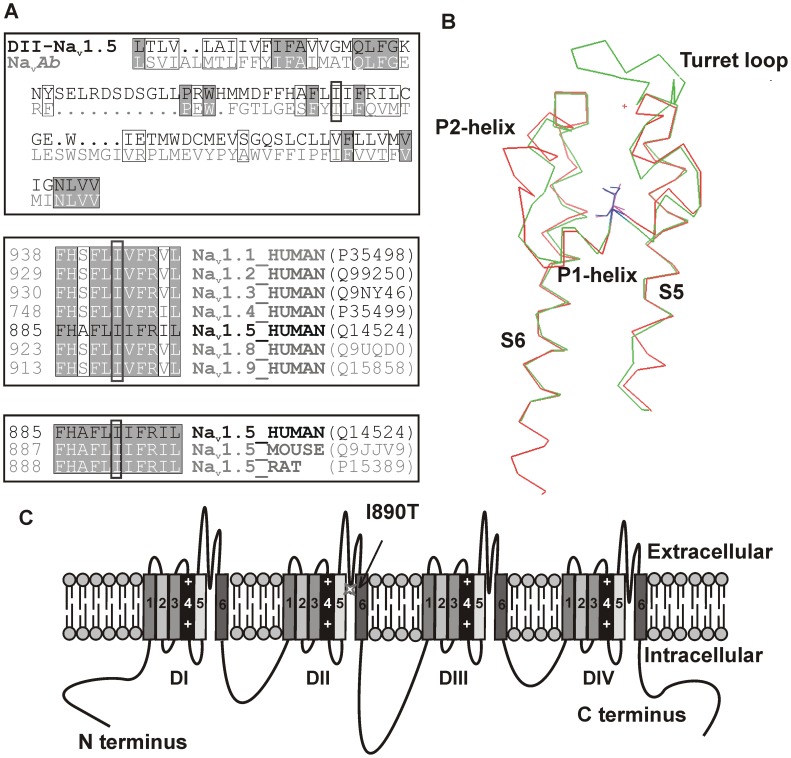
I890 is a conserved aminoacid, located in the intramembrane pore region of Na_v_1.5 DII. (A) Sequence alignment of the pore modules of human Na_v_1.5 channel (DII) and Na_v_ *Ab*. Identical aminoacids are highlighted in grey. Isoleucine-890 is marked with a dark box. Similar aminoacids are included inside light boxes and dots identify insertions (lower panel). Sequence alignment of human voltage-gated sodium channel α-subunit family members and of Na_v_1.5 channels of different species, upper and middle panels, respectively. The position of the first amino acid of each sequence is indicated on the left side, and the reference for each protein according to Uniprot is shown at the right side. (B) Partial view of the CPHmodel showing the pore module of DII of Na_v_1.5 channel (in green), based on the coordinates of Na_v_ *Ab* channel (in red). I890_Nav1.5_ and T169_Nav*Ab*_ are located in the middle of P1-helix and highlighted in blue and magenta, respectively. View from the interior side of the pore. (C) Na_v_1.5 channel scheme. The relative position of the I890T mutation in the S5–S6 loop of domain II (DII) is indicated with an arrow.

We performed a sequence alignment between DII of Na_v_1.5 channel and the corresponding sequence of Na_v_ *Ab* channel (Fig. 6A, upper panel) and built models for the S5–S6 region of the human channel using protein structure prediction models. Figure 6B shows the prediction obtained using the CPHmodels tool [20]. The loop S5–S6 in our model had a longer, flexible turret loop before the P1-helix. I890 resided within the second turn of the P1-helix, buried among the turret loop, the selectivity filter and the P2-helix.The structure of the S5–S6 loop was not apparently affected by the introduction of the I890T mutation (not shown).

## Discussion

In the present work we identified a novel *SCN5A* mutation in a patient diagnosed with BrS. The same mutation was found in three other family members, two of whom presented signs of potential arrhythmogenicity. Complete genetic analysis of the *SCN5A* gene revealed that the younger sister, who did not present any cardiac abnormalities, carried a common polymorphism (*p*.H558R), inherited from the father. This variation was not found in any of the other mutation carriers. One of the most intriguing features of BrS is the marked phenotypic variability. Clinical phenotype of individuals that carry *SCN5A* mutations may range from asymptomatic to SCD [26]. It has been postulated that modifying factors, such as genetic background and environment, influence the clinical phenotype of BrS patients [27], [28]. Several factors may explain the absence of symptoms in the younger sister. Young age and being a female are factors that diminish risk of arrhythmogenesis in BrS patients. In addition, the younger sister carries the polymorphism *p*.H558R which has been identified as a palliative factor in the pathological effects of BrS associated mutations [29], [30]. Still, this polymorphism was not found in the mother who, despite having suffered syncope, was not diagnosed with BrS. It is evident from these data that a combination of modifying factors is determinant of the clinical phenotype, especially when the functional effect of the pathogenic mutation is mild.

The *c*.2669 T>C nucleotidic change produces an amino acidic variation of an isoleucine-to-threonine in position 890 (*p*.Iso890Thr, *p*.I890T), localized in the P-loop of the domain II of the Na_v_1.5 channel (Fig. 6C). Sequence alignment demonstrated that I890 is conserved within the members of the voltage-gated sodium channel α-subunit family as well as in Na_v_1.5 channels of other species (Fig. 6A, middle and lower panels), indicating that a hydrophobic residue is essential at this position.

A correlation between the region of the Na_v_1.5 channel where mutations are located and the severity of the clinical phenotype has not been well established. Meregalli *et al.* [31] published a systematic study showing a correlation between the type of mutation and the changes produced in Na_v_1.5 currents. A broader study would most likely give light to this intriguing subject. Unfortunately, this type of study is difficult to achieve mainly due to the fact that only a small percentage of BrS related mutations are studied functionally. We have performed an exhaustive compilation of reported mutations related to BrS located in the pore regions of Na_v_1.5 channel (Table S1). Out of the 86 mutations identified in these regions, functional studies are documented only for 18 of them. The present work is the first reported functional study of a pore mutation in DII of Na_v_1.5 associated with BrS.

Our electrophysiological studies in transfected HEK cells showed that the mutation caused a significant decrease in current density compared to WT. In addition, the activation curve of the I890T currents was shifted to more positive potentials. It has been previously shown that mutations in the pore region may affect voltage activation of Na_v_1.5 and other Na_v_ channels [32]–[35]. The hypothesis that mutations in the pore region of Na_v_ channels can lead to structural perturbation compromising activation has been put forward [32]. In this context, our immunodetection experiments indicated that the decrease in *I*_Na_ observed in I890T cells could not be explained by a reduction in the membrane expression of the channel. Collectively, our observations support the idea that functional changes observed in the I890T currents are likely due to altered intrinsic properties of the channel.

The advent of the crystal structure of Na_v_ *Ab* [11] has provided a structural framework to model mutations in sodium channels. The homologous residue to I890Na_v_1.5 is T169Na_v_ *Ab* (Fig. 6A, upper panel). This Thr is conserved in the NaCh*Bac* bacterial sodium channel (T187NaCh*Bac*) and in the sodium channel of alphaproteobacteria HIMB114 (T172Na_v_ *Rh*), the structure of which has recently been solved [36]. In the available structure of Na_v_ *Ab* channel, T169Na_v_ *Ab* is buried within the intramembrane region of the pore. The polar group of T169Na_v_ *Ab* is stabilized by a hydrogen bond to R185Na_v_ *Ab* of the adjacent subunit [11]. The Na_v_ *Rh* channel stabilizes T172 in a similar fashion: the alcohol group of T172Na_v_ *Rh* is within hydrogen bond distance (2.7–3.2 Å depending on the subunit) of the carbonyl oxygens of R162 and I168 of the same chain. Interestingly, the mutation I890T recollects the bacterial residue at this position. However, in our I890T Na_v_1.5 model, no hydrogen bond acceptor candidate lies in the proximity of T890 and we cannot predict how the introduction of this polar group might be stabilized. It is interesting to note that isoleucine is the aminoacid present in position 890 in Na_v_1.5 as well as in the homologous position of different sodium channels in a wide variety of vertebrates (Figure S1). Thus, although the precise role of I890 in Na_v_1.5 is unknown, its presence at this position has been evolutionary favored.

In summary, we have identified a Na_v_1.5 pore mutation, I890T, in a BrS patient. This novel mutation causes an evident reduction in *I*_Na_ and a depolarizing shift in current voltage-dependent activation. Both mutation-dependent effects create the conditions for the observed pathophysiological manifestations of the patient. Although the observed changes in channel function in the mutated protein are mild, we cannot exclude that the effects of the mutation I890T in native myocytes could be different from those observed in our HEK cell experimental model, due to different regulatory factors. In addition, our functional study is analyzed in the context of the recently published crystal structure of the bacterial Na_v_ *Ab* and Na_v_ *Rh* channels. The evident, although mild, functional effect of the mutation correlates well with the lack of major structural changes found in the *in silico* analysis. In this sense, we believe that this type of studies hold the promise that correlations among channel structure, functional effects of mutations and clinical studies can lead to better understanding of SCD-related channelopathies.

### Limitations of the Structural Model

The most important limitation of our modelling approach was the use of 2 bacterial sodium channel structures (Na_v_ *Ab* and Na_v_ *Rh* resolution 2.7 and 3.05 Å, respectively) to model the pore domain of DII in Na_v_1.5. Amino acid sequences were only moderately conserved in S5–S6 of DII in Na_v_1.5 compared to Na_v_ *Rh* (17% identity and 25% homology) and Na_v_ *Ab* (22% identity and 46% homology). Consequently, at the resolution of the model, it was not straight-forward to anticipate the precise structural role of I890 in Na_v_1.5. We have tentatively proposed that the observed electrophysiological changes in I890T may be due to the introduction of the polar group of T890. This speculation was mostly based on the observation that T890 was stabilized by hydrogen bonds in bacterial channels, an interaction that was difficult to envision in I890T Na_v_1.5 models. We acknowledge that this is an indirect argument. We believe, though, that our *in silico* analyses as well as the alignment data presented here support the idea that, in the absence of a neighboring hydrogen donor, an isoleucine may be more appropriate at that position.

## Supporting Information

Figure S1 **I890 is a highy conserved aminoacid among vertebrates.** Sequence alignment of voltage-gated sodium channel α-subunit family members of different species. Human Na_v_1.5 I890 and its homologues are marked with a dark box. Identical amimoacids are highlighted in grey. Similar aminoacids are included inside light boxes.
(TIF)

Table S1 Reported *SCN5A* mutations related to Brugada Syndrome in pore regions of Na_v_1.5. The table contains all missense and nonsense mutations reported in the Human Gene Mutation Database (HGMD) Professional (version 2012.1 from 30/03/2012) [21] and in the repository of genetic data on the inherited arrhythmogenic diseases [61]. The mutation sites and aminoacid changes are indicated, together with the Na_v_1.5 pore domain where they are localized, and the main results of the electrophysiological studies, when performed. Not performed (NP) indicates that no functional studies have been reported.
(DOC)
