# Supplementary figures and images for: A Novel Missense Mutation, I890T, in the Pore Region of Cardiac Sodium Channel Causes Brugada Syndrome
Source: PLoS One. 2013 Jan 7;8(1):e53220. doi: 10.1371/journal.pone.0053220 (PMC3538753; doi:10.1371/journal.pone.0053220)

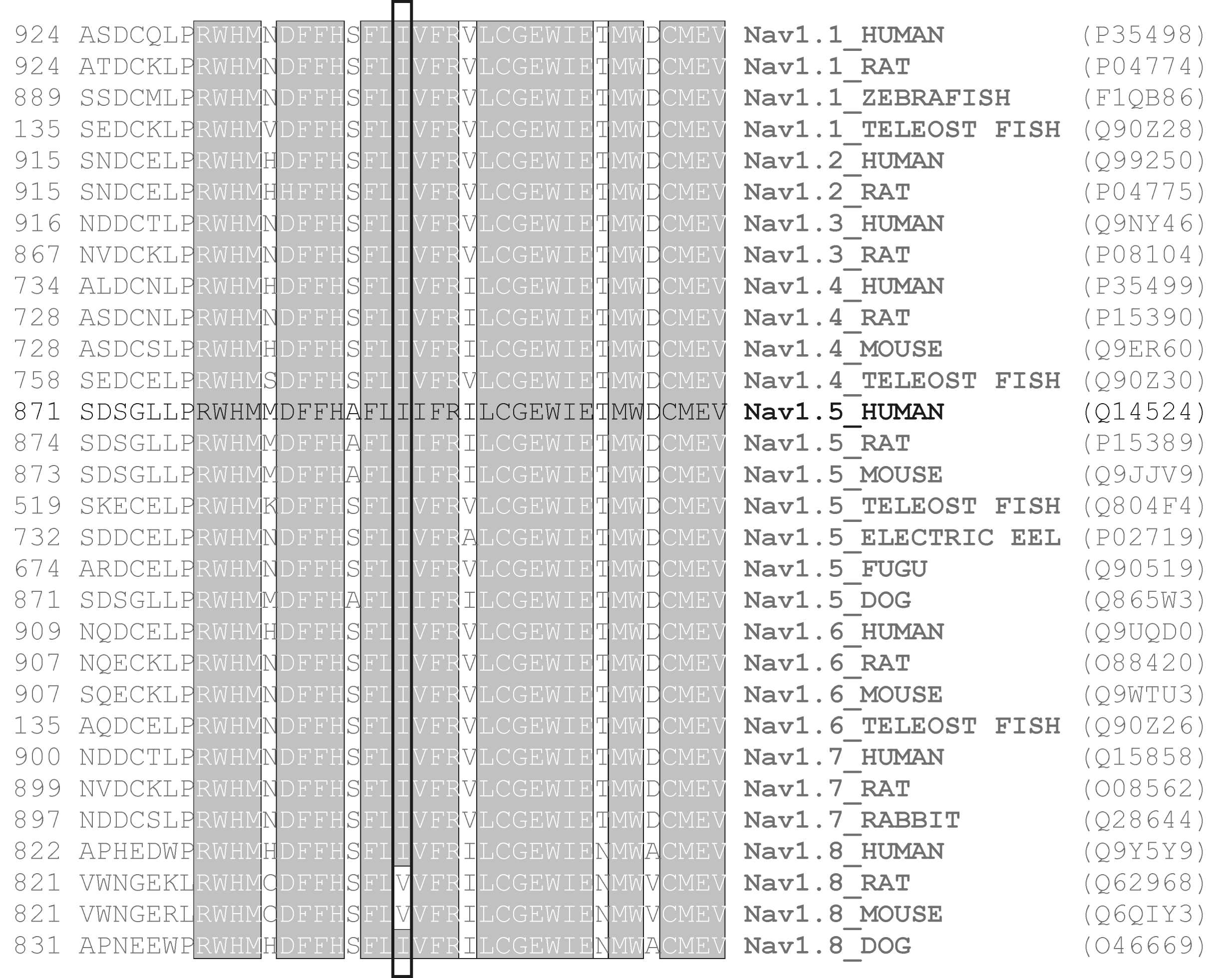

Supplement: Figure S1 — I890 is a highy conserved aminoacid among vertebrates. Sequence alignment of voltage-gated sodium channel α-subunit family members of different species. Human Nav1.5 I890 and its homologues are marked with a dark box. Identical amimoacids are highlighted in grey. Similar aminoacids are included inside light boxes. (TIF) [file pone.0053220.s001.tif]
