# Supplementary material for: A Novel Missense Mutation, I890T, in the Pore Region of Cardiac Sodium Channel Causes Brugada Syndrome
Source: PLoS One. 2013 Jan 7;8(1):e53220. doi: 10.1371/journal.pone.0053220 (PMC3538753; doi:10.1371/journal.pone.0053220)
Supplement: Table S1 — Reported SCN5A mutations related to Brugada Syndrome in pore regions of Nav1.5. The table contains all missense and nonsense mutations reported in the Human Gene Mutation Database (HGMD) Professional (version 2012.1 from 30/03/2012) [21] and in the repository of genetic data on the inherited arrhythmogenic diseases [61]. The mutation sites and aminoacid changes are indicated, together with the Nav1.5 pore domain where they are localized, and the main results of the electrophysiological studies, when performed. Not performed (NP) indicates that no functional studies have been reported. (DOC) [file pone.0053220.s002.doc]

**Table S1. Reported *SCN5A* mutations related to Brugada Syndrome in pore regions of Na_v_1.5.**

| **Mutation** | **Na_V_1.5 Domain** | **Electrophysiological Studies** | **Reference** |
| --- | --- | --- | --- |
| H278D | DI | NP | Kapplinger, 2010 [6] |
| R282H | DI | *- I*_Na_; mild positive shift in activation | Itoh, 2005 [33] |
| R282C | DI | NP | Kapplinger, 2010 [6] |
| G292S | DI | NP | Niimura, 2004 |
| V294M | DI | NP | Priori, 2002 |
| V300I | DI | NP | Kapplinger, 2010 [6] |
| W301X | DI | NP | Kotta, 2010 |
| L315P | DI | NP | Kapplinger, 2010 [6] |
| K317N | DI | Report plasmid generation | Yi, 2003 |
| G319S | DI | NP | Priori, 2002 |
| T320N | DI | NP | Kapplinger, 2010 [6] |
| L325R | DI | *- I*_Na_; positive shift in activation; slower time to peak and onset of fast inactivation | Keller, 2005 [34] |
| P336L | DI | - *I*_Na_ | Cordeiro, 2006 |
| E346X | DI | NP | Meregalli, 2009 [31] |
| G351V | DI | *- I*_Na_ | Vatta, 2002 |
| G351D | DI | NP | Kapplinger, 2010 [6] |
| T353I | DI | *- I*_Na_; negative shift in inactivation; trafficking defect | Pfahnl, 2007 |
| D356N | DI | No current | Makiyama, 2005 |
| R367C | DI | No current | Meregalli, 2009 [31] |
| R367L | DI | NP | Kapplinger, 2010 [6] |
| R367H | DI | No current | Vatta, 2002 ;  Hong, 2004 |
| M369K | DI | NP | Smits, 2002 |
| W374G | DI | NP | Kapplinger, 2010 [6] |
| R376H | DI | *- I*_Na_ | Rossenbacker, 2004 |
| G386R | DI | NP | Kapplinger, 2010 [6] |
| G386E | DI | NP | Kapplinger, 2010 [6] |
| E867Q | DII | NP | Kapplinger, 2010 [6] |
| L867X | DII | NP | Smits, 2002 |
| L868X | DII | NP | Kapplinger, 2010 [6] |
| S871fs+9X | DII | NP | Priori, 2002 |
| E876Q | DII | NP | Kapplinger, 2010 [6] |
| R878H | DII | NP | Kapplinger, 2010 [6] |
| R886P | DII | NP | Kapplinger, 2010 [6] |
| **I890T** | **DII** | ***- I*_Na_; positive shift in activation** | **Present work** |
| F892I | DII | NP | Priori, 2002 |
| R893C | DII | NP | Kapplinger, 2010 [6] |
| R893H | DII | NP | Kapplinger, 2010 [6] |
| C896S | DII | NP | Priori, 2002 |
| E901K | DII | NP | Kapplinger, 2010 [6] |
| S910L | DII | NP | Priori, 2002 |
| F1360C | DIII | NP | Kapplinger, 2010 [6] |
| C1363Y | DIII | NP | Meregalli, 2006 [31] |
| L1373X | DIII | NP | Kapplinger, 2010 [6] |
| S1382I | DIII | NP | Smits, 2002 |
| Q1383X | DIII | NP | Kapplinger, 2010 [6] |
| L1393X | DIII | No current | Samani, 2009 |
| Y1394X | DIII | NP | Kapplinger, 2010 [6] |
| K1397RfsX2 | DIII | NP | Kapplinger, 2010 [6] |
| V1405M | DIII | NP | Kapplinger, 2010 [6] |
| V1405L | DIII | NP | Smits, 2002 |
| G1406R | DIII | No current | Kindt, 2001 |
| V1406E | DIII | NP | Kapplinger, 2010 [6] |
| G1408R | DIII | NP | Meregalli, 2009 [31] |
| Y1409C | DIII | NP | Kapplinger, 2010 [6] |
| Y1409X | DIII | NP | Kapplinger, 2010 [6] |
| L1412F | DIII | NP | Kapplinger, 2010 [6] |
| K1419E | DIII | NP | Kapplinger, 2010 [6] |
| G1420R | DIII | NP | Kapplinger, 2010 [6] |
| G1420V | DIII | NP | Hermida, 2010 |
| A1427S | DIII | NP | Kapplinger, 2010 [6] |
| A1428V | DIII | NP | Kapplinger, 2010 [6] |
| R1432S | DIII | NP | Kapplinger, 2010 [6] |
| R1432G | DIII | No current //  No current; trafficking defect | Deschenes, 2000 // Baroudi, 2001 |
| G1433V | DIII | NP | Kapplinger, 2010 [6] |
| G1433G | DIII | NP | Kapplinger, 2010 [6] |
| Y1434X | DIII | NP | Kapplinger, 2010 [6] |
| P1438L | DIII | No current | Six, 2008 |
| W1440X | DIII | NP | Kapplinger, 2010 [6] |
| E1441Q | DIII | NP | Kapplinger, 2010 [6] |
| D1690Hfsx98 | DIV | NP | Kapplinger, 2010 [6] |
| Q1695X | DIV | NP | Meregalli, 2009 [31] |
| A1698T | DIV | NP | Kapplinger, 2010 [6] |
| Q1706H | DIV | NP | Nakajima, 2011 |
| T1709del | DIV | NP | Kapplinger, 2010 [6] |
| T1709M | DIV | NP | Yokokawa, 2007 |
| T1709R | DIV | NP | Kapplinger, 2010 [6] |
| S1710L | DIV | Positive shift in activation; negative shift in inactivation; defects in inactivation kinetics | Shirai, 2002 [35] |
| G1712S | DIV | NP | Kapplinger, 2010 [6] |
| D1714G | DIV | *- I*_Na_ | Amin, 2005 |
| P1719fsX67 | DIV | NP | Yokokawa, 2007 |
| I1720SfsX67 | DIV | NP | Kapplinger, 2010 [6] |
| N1722D | DIV | NP | Probst, 2009 [27] |
| C1728R | DIV | NP | Kapplinger, 2010 [6] |
| C1728W | DIV | NP | Kapplinger, 2010 [6] |
| G1740R | DIV | No current; trafficking defect | Baroudi, 2004 |
| G1743E | DIV | *- I*_Na_ | Vernooy, 2006 |
| G1743R | DIV | No current; trafficking defect | Valdivia, 2004 |

**Supplemental references.**

37. Niimura H, Matsunaga A, Kumagai K, Ohwaki K, Ogawa M, et al. [2004] Genetic analysis of Brugada syndrome in Western Japan: two novel mutations. Circ J 68: 740-746.

38. Priori SG, Napolitano C, Gasparini M, Pappone C, Della Bella P, et al. [2002] Natural history of Brugada syndrome: insights for risk stratification and management. Circulation 105: 1342-1347.

39. Kotta CM, Anastasakis A, Gatzoulis K, Manolis AS, Stefanadis C [2010] Novel sodium channel SCN5A mutations in Brugada syndrome patients from Greece. Int J Cardiol 145: 45-48.

40. Yi SD, Meng SR, Cui YK, Chen ZM, Peng J [2003] [PCR-based site-directed mutagenesis and recombinant expression plasmid construction of a SCN5A mutation [K317N] identified in a Chinese family with Brugada syndrome]. Di Yi Jun Yi Da Xue Xue Bao 23: 1139-1142.

41. Cordeiro JM, Barajas-Martinez H, Hong K, Burashnikov E, Pfeiffer R, et al. [2006] Compound heterozygous mutations P336L and I1660V in the human cardiac sodium channel associated with the Brugada syndrome. Circulation 114: 2026-2033.

42. Vatta M, Dumaine R, Antzelevitch C, Brugada R, Li H, et al. [2002] Novel mutations in domain I of SCN5A cause Brugada syndrome. Mol Genet Metab 75: 317-324.

43. Pfahnl AE, Viswanathan PC, Weiss R, Shang LL, Sanyal S, et al. [2007] A sodium channel pore mutation causing Brugada syndrome. Heart Rhythm 4: 46-53.

44. Makiyama T, Akao M, Tsuji K, Doi T, Ohno S, et al. [2005] High risk for bradyarrhythmic complications in patients with Brugada syndrome caused by SCN5A gene mutations. J Am Coll Cardiol 46: 2100-2106.

45. Vatta M, Dumaine R, Varghese G, Richard TA, Shimizu W, et al. [2002] Genetic and biophysical basis of sudden unexplained nocturnal death syndrome [SUNDS], a disease allelic to Brugada syndrome. Hum Mol Genet 11: 337-345.

46. Hong K, Berruezo-Sanchez A, Poungvarin N, Oliva A, Vatta M, et al. [2004] Phenotypic characterization of a large European family with Brugada syndrome displaying a sudden unexpected death syndrome mutation in SCN5A. J Cardiovasc Electrophysiol 15: 64-69.

47. Smits JP, Eckardt L, Probst V, Bezzina CR, Schott JJ, et al. [2002] Genotype-phenotype relationship in Brugada syndrome: electrocardiographic features differentiate SCN5A-related patients from non-SCN5A-related patients. J Am Coll Cardiol 40: 350-356.

48. Rossenbacker T, Carroll SJ, Liu H, Kuiperi C, de Ravel TJ, et al. [2004] Novel pore mutation in SCN5A manifests as a spectrum of phenotypes ranging from atrial flutter, conduction disease, and Brugada syndrome to sudden cardiac death. Heart Rhythm 1: 610-615.

49. Samani K, Ai T, Towbin JA, Brugada R, Shuraih M, et al. [2009] A nonsense SCN5A mutation associated with Brugada-type electrocardiogram and intraventricular conduction defects. Pacing Clin Electrophysiol 32: 1231-1236.

50. Kyndt F, Probst V, Potet F, Demolombe S, Chevallier JC, et al. [2001] Novel SCN5A mutation leading either to isolated cardiac conduction defect or Brugada syndrome in a large French family. Circulation 104: 3081-3086.

51. Hermida JS, Dassonvalle E, Six I, Amant C, Coviaux F, et al. [2010] Prospective evaluation of the familial prevalence of the brugada syndrome. Am J Cardiol 106: 1758-1762.

52. Deschenes I, Baroudi G, Berthet M, Barde I, Chalvidan T, et al. [2000] Electrophysiological characterization of SCN5A mutations causing long QT [E1784K] and Brugada [R1512W and R1432G] syndromes. Cardiovasc Res 46: 55-65.

53. Baroudi G, Pouliot V, Denjoy I, Guicheney P, Shrier A, et al. [2001] Novel mechanism for Brugada syndrome: defective surface localization of an SCN5A mutant [R1432G]. Circ Res 88: E78-83.

54. Six I, Hermida JS, Huang H, Gouas L, Fressart V, et al. [2008] The occurrence of Brugada syndrome and isolated cardiac conductive disease in the same family could be due to a single SCN5A mutation or to the accidental association of both diseases. Europace 10: 79-85.

55. Nakajima T, Kaneko Y, Saito A, Irie T, Tange S, et al. [2011] Identification of six novel SCN5A mutations in Japanese patients with Brugada syndrome. Int Heart J 52: 27-31.

56. Yokokawa M, Noda T, Okamura H, Satomi K, Suyama K, et al. [2007] Comparison of long-term follow-up of electrocardiographic features in Brugada syndrome between the SCN5A-positive probands and the SCN5A-negative probands. Am J Cardiol 100: 649-655.

57. Amin AS, Verkerk AO, Bhuiyan ZA, Wilde AA, Tan HL [2005] Novel Brugada syndrome-causing mutation in ion-conducting pore of cardiac Na+ channel does not affect ion selectivity properties. Acta Physiol Scand 185: 291-301.

58. Baroudi G, Napolitano C, Priori SG, Del Bufalo A, Chahine M [2004] Loss of function associated with novel mutations of the SCN5A gene in patients with Brugada syndrome. Can J Cardiol 20: 425-430.

59. Vernooy K, Sicouri S, Dumaine R, Hong K, Oliva A, et al. [2006] Genetic and biophysical basis for bupivacaine-induced ST segment elevation and VT/VF. Anesthesia unmasked Brugada syndrome. Heart Rhythm 3: 1074-1078.

60. Valdivia CR, Tester DJ, Rok BA, Porter CB, Munger TM, et al. [2004] A trafficking defective, Brugada syndrome-causing SCN5A mutation rescued by drugs. Cardiovasc Res 62: 53-62.

61. Repository of genetic data on the inherited arrhythmogenic diseases website. Available: http://www.fsm.it/cardmoc/. Acessed 2012 July.
